# P-633. Burden of school absenteeism and medical visits in pre-kindergarden-12th grade students and staff with acute respiratory illnesses

**DOI:** 10.1093/ofid/ofaf695.846

**Published:** 2026-01-11

**Authors:** Jennifer Goldman, Brian R Lee, Hannah L Kirking, Brittney Fritschmann, Nibha Sagar, Dithi Banerjee, Anjana Sasidharan, Rangaraj Selvarangan, Olivia Almendares, Jennifer E Schuster

**Affiliations:** Children's Mercy Hospital, Kansas City, MO; Children's Mercy Kansas City, Kansas City, Missouri; Coronavirus and Other Respiratory Viruses Division, National Center for Immunization and Respiratory Diseases, CDC, Atlanta, GA; Children's Mercy Hospital, Kansas City, MO; Children's Mercy hospital, Kansas City, Missouri; Children's Mercy Hospital, Kansas City, MO; Childrens Mercy Hospital, Missouri, Kansas; Children’s Mercy Hospital, Kansas City, Missouri; Centers for Disease Control and Prevention, Atlanta, Georgia; Children's Mercy Kansas City, Kansas City, Missouri

## Abstract

**Background:**

Acute respiratory illnesses (ARI) are common and their contribution to school absenteeism is poorly understood. Using school-based respiratory virus surveillance data, we examined viral detections, missed school/work, and the association between absenteeism and viral detections in students and staff with ARI.

**Table.**
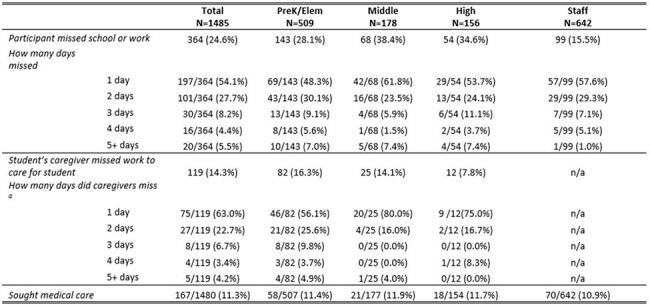
Survey-reported (n=1485) ARI-associated absenteeism and medical visits of School KIDS participants during the 2024-25 school year

**Figure.**
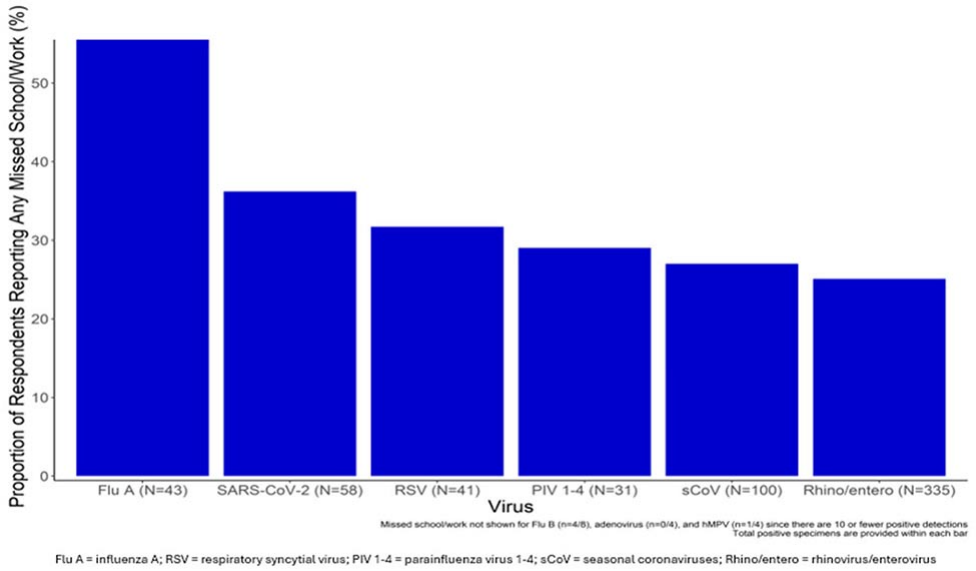
Proportion of respondents with ARI and a positive viral detection who missed school/work during the 2024-25 school year

**Methods:**

School KIDS is a prospective respiratory virus surveillance program in a Kansas City, MO preK–12^th^ grade public school district. Enrolled students and staff provide monthly anterior nasal swabs at school and can demand testing when experiencing ARI symptoms. Participants complete surveys within 24 hours before (‘pre-test’) and 7 days after (‘post-test') specimen collection. Surveys capture ARI symptoms (cough, fever, nasal congestion, shortness of breath, runny nose, wheezing, sore throat); school and work absenteeism for students, staff, and caretakers; and medical care. Analysis included participants with ARI symptoms on the pre-test survey and a completed post-test survey. Specimens were tested by PCR for respiratory syncytial virus (RSV), influenza (Flu), seasonal coronaviruses (sCoV), parainfluenza virus, adenovirus, rhinovirus/enterovirus (RV/EV), human metapneumovirus, and SARS-CoV-2.

**Results:**

From September 2024-March 2025, 656 participants (preK/elementary=229, middle=83, high=75, staff=269) submitted 1,485 pre-test surveys noting ARI symptoms and completed corresponding post-test surveys. Overall, 25% (364/1485) reported missing ≥1 day of school or work, and 11% reported seeking medical care (Table). Fourteen percent of caregivers missed work to care for a student. Nearly 40% (589/1485) of specimens had ≥1 detected virus; the most common were RV/EV (n=335), sCoV (n=100), SARS-CoV-2 (n=58), Flu A (n=43), and RSV (n=41). Participants with symptoms and a virus were more likely to report absence (29%) compared to those with symptoms but no virus (22%; P < 0.001, Pearson’s chi-square). Absenteeism was most common in those with Flu A (24/43), SARS-CoV-2 (21/58), and RSV (13/41) (Figure).

**Conclusion:**

School absenteeism was frequent due to ARI. Additional strategies are needed to keep students and staff healthy to minimize ARI associated absenteeism and help optimize educational outcomes.

**Disclosures:**

Brian R. Lee, PhD, MPH, Merck: Grant/Research Support Rangaraj Selvarangan, PhD, Altona: Grant/Research Support|Biomerieux: Advisor/Consultant|Biomerieux: Grant/Research Support|Biomerieux: Honoraria|Cepheid: Grant/Research Support|Hologic: Grant/Research Support|Hologic: Honoraria|Meridian: Grant/Research Support|Qiagen: Grant/Research Support

